# The effects of kangaroo mother care on the time to breastfeeding initiation among preterm and LBW infants: a meta-analysis of published studies

**DOI:** 10.1186/s13006-019-0206-0

**Published:** 2019-02-19

**Authors:** Alemayehu Gonie Mekonnen, Sisay Shewasinad Yehualashet, Alebachew Demelash Bayleyegn

**Affiliations:** Department of Nursing, College of Health Sciences, Debre Berhan University, Debre Berhan, Ethiopia

**Keywords:** Kangaroo mother care, Initiation of breastfeeding, Preterm and LBW infants

## Abstract

**Background:**

Kangaroo mother care is a comprehensive intervention given for all newborns especially for premature and low birthweight infants. It is the most feasible and preferred intervention for decreasing neonatal morbidity and mortality. Even though time to initiating breastfeeding has been examined by randomized controlled trials, varying findings have been reported. Therefore, the main objective of this meta-analysis was to estimate the pooled mean time to initiate breastfeeding among preterm and low birthweight infants.

**Methods:**

The authors searched for randomized controlled trial studies conducted on the effects of kangaroo mother care on the time to breastfeeding initiation among preterm and low birthweight infants. Published articles were identified through a computerized search of electronic databases that includes MEDLINE via PubMed, EMBASE, CINAHL and CENTRAL. The search terms were kangaroo mother care or (skin to skin), or conventional care, newborns, preterm infants, low birthweight infants and randomized controlled trial. A total of 467 eligible titles were identified and eight studies met the inclusion criteria. The extracted data were entered and analyzed using Cochrane Review Manager-5-3 software. Heterogeneity across studies was evaluated by Chi^2^ test and inconsistency index (I^2^). Publication bias was assessed using a funnel plot. The random effect model was applied to estimate the pooled mean time to initiate breastfeeding with 95% confidence interval.

**Results:**

In this meta-analysis, the overall pooled mean time to initiate breastfeeding was 2.6 days (95% CI 1.23, 3.96). Preterm and low birthweight infants receiving kangaroo mother care intervention initiated breastfeeding 2 days 14 h 24 min earlier than conventional care of radiant warmer/incubator method.

**Conclusions:**

Kangaroo mother care promotes early initiation of breastfeeding as compared to conventional care method. Therefore, health facilities need to implement the kangaroo mother care for preterm and low birthweight infants.

**Electronic supplementary material:**

The online version of this article (10.1186/s13006-019-0206-0) contains supplementary material, which is available to authorized users.

## Background

Kangaroo mother care (KMC) was first started in Colombia in 1978 [1]. It is a comprehensive intervention given for all newborns especially for premature and low birthweight (LBW) infants. Kangaroo mother care is the most feasible, readily available, and preferred intervention for decreasing neonatal morbidity and mortality in developed and developing countries, and suitable for use in all settings [2]. It is based on three components: kangaroo position (skin-to-skin contact between mother and infant), breastfeeding; and timely discharge with close follow-up [3]. This procedure could be done at any time during the night and day [4] and creates an optimal environment for adaptation of newborn infants to extra-uterine life [5].

Skin-to-skin contact between the mother and the baby is a safe and inexpensive procedure that has proven benefits for mothers and children as compared to an incubator caring method. It plays a significant role on infant survival, neurodevelopment, and the quality of mother-infant bonding. Kangaroo mother care complements good quality care and allows providers to ration use of expensive resources such as warmers and incubators [6, 7].

In preterm and LBW infants, skin-to-skin contact between the mother and her infant decreases maternal postpartum depressive symptoms [8] and improves self-efficacy and mother-child bonding [9]. Preterm and LBW infants who are receiving KMC gain more weight per day, have better heart rate and breathing regulation, and have effective oxygenation preterm [4, 10]. Furthermore, it facilitates the newborn early initiation and effective breastfeeding [11], and in turn effective breastfeeding reduces the incidence of necrotizing enterocolitis which is a leading cause of death in preterm infants [12, 13].

Kangaroo mother care for preterm infants is also related to better cognitive and motor development at six months of age [8]. Other than the neonatal and maternal health benefits, KMC is an important tool in reducing the postpartum hospital stay, thereby cutting down the overall healthcare expenditure and provides economic benefit to the parents [7]. Preterm and LBW infants who were given KMC spent less time in the hospital than those who were given standard care [14]. Even though time to initiating breastfeeding was examined by randomized control trial studies, varying findings were stated. Therefore, the main objective of this meta-analysis was to estimate the pooled mean time to initiate breastfeeding among preterm and low birthweight infants.

### Hypothesis

There is a mean time difference between kangaroo mother care and the conventional care method to initiate breastfeeding among preterm and LBW infants. Preterm and LBW infants receiving KMC have earlier initiation of breastfeeding than conventional care (radiant warmer/incubator).

## Methods

This review covered all randomized controlled trial studies of kangaroo mother care versus conventional care method and its breastfeeding initiation among premature and LBW infants. The search strategy focused on human’s category and included randomized controlled trial studies published from January 2000 to June 30, 2018. The review authors searched the studies using the following databases: MEDLINE via PubMed, EMBASE, CINAHL (Cumulative Index to Nursing and Allied Health Literature) and CENTRAL (Cochrane Central Register of Controlled Trials). The search terms were KMC or (skin to skin), or conventional care method, newborns, preterm infants, LBW infants, randomized controlled trial (Additional file 1). These key terms were combined using Boolean operators “AND” and “OR” to narrow the search. In addition, we searched reference lists of identified studies, peer reviewed articles, and Google Scholar to augment database searches. To identify relevant articles, titles and abstracts of retrieved articles were exported to Endnote to screen duplicate articles. Then three review authors assessed and reviewed independently all studies deemed suitable to determine inclusion.

### Study selection criteria

- Inclusion criteria: To meet inclusion criteria, titles and abstracts of studies were examined, and the following inclusion criteria were included
  - Full text randomized controlled trial study design
  - Studies published after year 2000 (to minimize the time lag bias)
  - Peer-reviewed and published in the English language
  - Reported the mean time and standard deviations to initiate exclusive breastfeeding
- Exclusion criteria: The following exclusion criteria were used in screening articles for the current meta-analysis:
  - Reported the knowledge and practice of mothers towards KMC
  - Studies that repeated findings from the already included studies
  - Studies of KMC on the normal infants

### Study selection and data extraction

The authors screened all identified titles and abstracts. Articles found relevant by title and abstract were undergone for full-text review for eligibility. For eligible studies, three authors extracted the data using the pre-determined inclusion criteria. The review authors recorded the data on Microsoft excel and extracted the following data for each trial: the authors, year of publication, study setting, study design, sample size, and inclusion criteria (Table 1). The study selection and data extraction were done from April 10 to June 30, 2018. Data extraction was performed independently by three review authors (AG, SS and AD) using a pre-specified data extraction form. Discrepancies were solved through discussion and articles were included after consensus was reached. The authors communicated the trial authors for clarification.

**Table 1:** Summary of RCT studies included in the analysis, June 30, 2018

Sr.	Author	Publication year	KMC mean time to initiate breastfeeding			Radiator warmer			Study design	Inclusion criteria	Setting
			mean	SD	Total	mean	SD	Total			
1	Rahman et al. [18]	2017	9.10	2.40	40	14.70	4.50	40	RCT	Preterm + LBW	Bangladesh
2	Mahbubul et al. [17]	2017	9.53	3.35	40	14.35	6.06	40	RCT	LBW	Bangladesh
3	Lamy Filho et al. [16]	2008	18.90	11.4	354	24.10	12.8	609	RCT	LBW	Brazil
4	Sharma et al. [20]	2016	10.10	5.90	71	10.80	4.30	70	RCT	Preterm + LBW	India
5	Suman Rao et al. [22]	2008	5.71	5.65	103	4.85	4.94	103	RCT	LBW	India
6	Ghavane et al. [21]	2012	9.70	6.40	71	11.00	8.10	69	RCT	LBW	India
7	Jagadale & Salunkhe [15]	2014	5.4	1.10	25	6.7	2.92	25	RCT	LBW	India
8	Srivastava et al. [19]	2014	6.71	1.89	118	9.55	1.14	122	RCT	Preterm	India

### Data analysis

The extracted data were entered and analyzed using a Cochrane review manager-5-3 software. Potential sources of heterogeneity across studies was evaluated by Chi^2^ test which verifies the presence of heterogeneity, and inconsistency index (I^2^) which describes the percentage of total variation across studies. The I^2^ provides the percentage of variability due to heterogeneity rather than the chance difference or sampling error. The I^2^ greater than 75% and Chi^2^ test (*p* < 0.10) was considered statistically significant heterogeneity [13]. The random effects model which assesses the variability within and between studies was applied to estimate the pooled mean time to initiate exclusive breastfeeding with 95% confidence interval.

Publication bias was assessed using the funnel plot (which displays effect sizes plotted against the sample size, standard error, conditional variance, or some other measure of the precision of the estimate). In the presence of a cloud of data points that is symmetric around the population effect size and has the shape of a funnel, one can conclude as no publication bias [13]. Sensitivity analysis was performed to explore the effects of random-effects analyses for primary outcomes with statistical heterogeneity. In addition, to be free of other sources of bias, review authors independently assessed risk of bias in included studies and resolved discrepancies through discussion.

## Results

### Characteristics of included studies

**Fig. 1:**
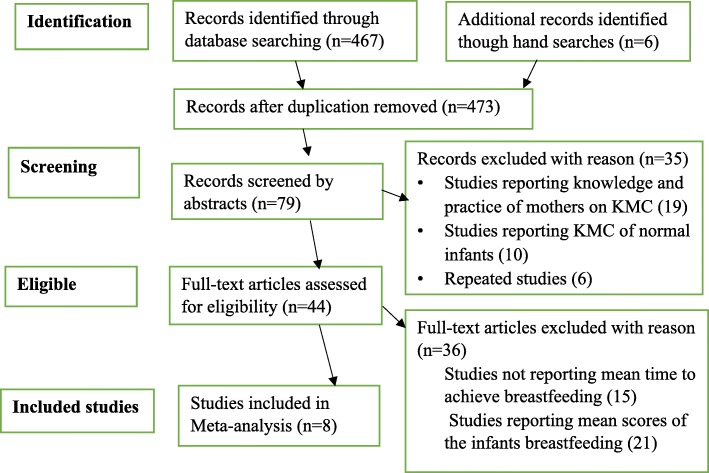
PRISMA flow-diagram that depicts the phases of study selection

Totally, 467 studies were retrieved, of which 44 were identified as being potentially eligible for this review. Altogether, eight randomized control trial studies (Table 1) comprising of 1900 participants were included in the meta-analysis (Fig. 1). All included articles were facility-based studies and conducted in neonatal care and postnatal care units. The studies included preterm and LBW neonates with a sample size ranging from 50 [15] to 963 [16]. Among the articles included in the analysis, seven studies reported that neonate in the KMC group initiated exclusive breastfeeding earlier than the conventional care neonates [15–21]. On the contrary, one study reported that conventional care neonate initiated breastfeeding earlier than KMC neonates [22]. The studies were conducted in India [15, 19–22], Brazil [16], Bangladesh [17, 18].

### Heterogeneity and publication bias

**Fig. 2:**
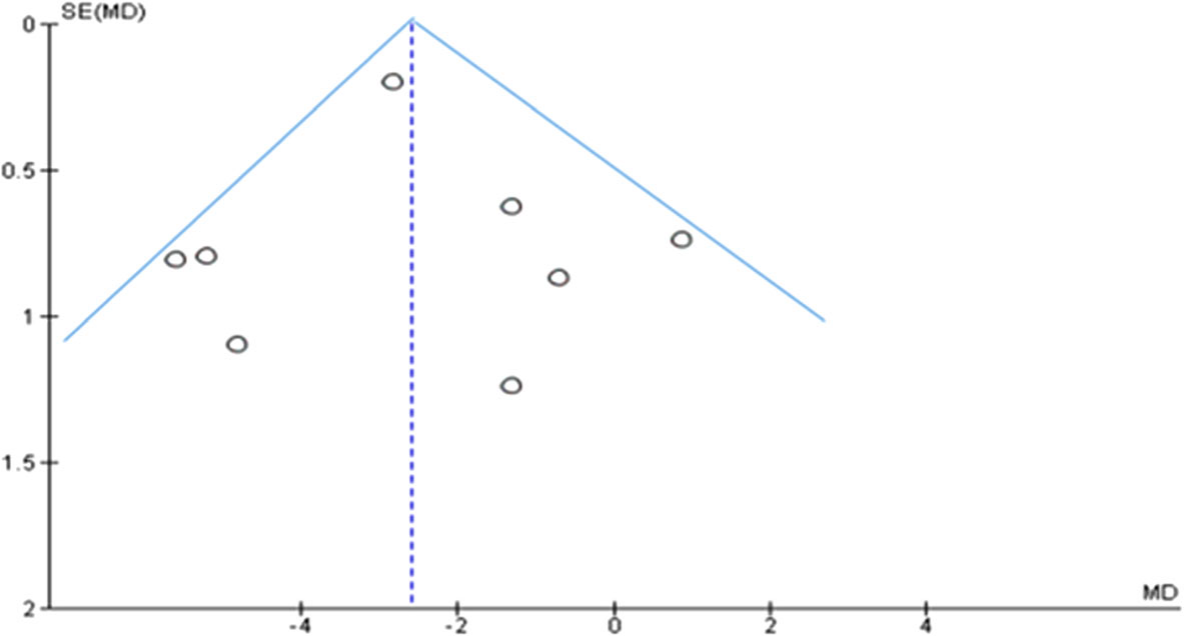
Funnel plot showing publication bias (inverted symmetrical funnel plot)

The included studies were assessed for heterogeneity and publication bias. Accordingly, the analysis showed a substantial heterogeneity among studies with Chi^2^ = 61.78; *p* < 0.00001 and I^2^ statistics (I^2^ = 89%). The publication bias was checked by a funnel plot, and the plot has a symmetric inverted funnel shape showing no evidence of variability in effect sizes from studies and publication bias (Fig. 2).

### Sensitivity analysis

Sensitivity analysis of the eight studies were performed to test the effect of each study on the pooled result and no statistical source of heterogeneity among the eight studies were identified.

### The pooled mean time to initiate breastfeeding

**Fig. 3:**
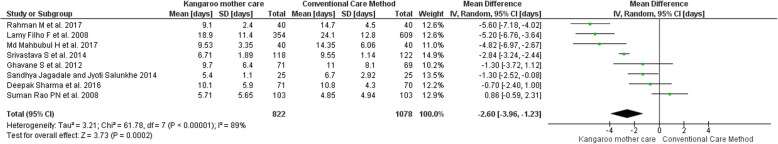
Forest plot of pooled mean time difference to initiate exclusive breastfeeding among preterm and LBW infants

The pooled mean time to initiate breastfeeding is presented in a forest plot (Fig. 3). The reported mean time to initiate breastfeeding in each study ranged from around half day [20] to 5 days [18] with a substantial heterogeneity across studies (Chi^2^ = 61.78; *p* < 0.00001; I^2^ = 89%). In this meta-analysis, the overall pooled mean time for initiating breastfeeding from the random effects model revealed 2.6 days (2 days 14 h 24 min) (95% CI 1.23, 3.96). This indicated that preterm and low birthweight infants in the KMC intervention group initiated exclusive breastfeeding 2.6 days earlier than conventional care method (Fig. 3).

## Discussion

A systematic review of randomized controlled trials that comparing KMC and conventional neonatal care found compelling evidences which support KMC could improve breastfeeding rates in high-income countries in which conventional neonatal care is unavailable [10, 23]. In this meta-analysis, preterm and low birthweight infants in the KMC intervention group initiated breastfeeding 2.6 days earlier than conventional care method. This is in line with the previous systematic review studies in which KMC group initiated breastfeeding 1.6 days earlier than conventional neonatal care [10]. It was observed that infants who exposed to KMC showed significantly better emotion regulation than infants who exposed to the usual standard care which again initiated early breastfeeding [24, 25]. Furthermore, KMC has a significant role in starting breastfeeding among preterm and LBW infants [26–28]. Sloan NL et al. also reported that women in the community kangaroo mother care group initiated to breastfeed earlier than the control group [29]. This confirms the conclusion that KMC promotes early initiation of breastfeeding as compared to conventional care methods.

In this meta-analysis, preterm and LBW infants, initiated breastfeeding earlier than the conventional care group. Similarly, Moore ER et al. in their systematic review of randomized controlled studies, concluded that KMC improved the likelihood of breastfeeding initiation [30]. Various studies have also reported higher breastfeeding rates among KMC group as compared with conventional care method [28, 31, 32]. This could be explained by kangaroo mother care having a better effect on thermal regulation and reduced levels of infant stress, and is more comfortable than conventional neonatal care.

### Limitations

In this analysis, the included studies were limited in number (only eight studies) which did not enable us to determine an effect size on all outcomes. The study searched only English language reports. In addition, this study was based only on published peer-reviewed studies and important data might be missed from unpublished studies.

## Conclusions

Women in the kangaroo mother care intervention group, initiated breastfeeding earlier than conventional care method. Kangaroo mother care promotes early initiation of breastfeeding as compared to conventional care method. Therefore, health facilities need to implement the kangaroo mother care for preterm and low birthweight infants.

## Additional file

Additional file 1: MEDLINE via PubMed and Cochrane database search for randomized controlled trial studies of KMC versus conventional care method, June 30, 2018. (DOCX 150 kb)

## Abbreviations

KMC: Kangaroo mother care
LBW: Low birthweight
RCT: Randomized controlled trial
