# Supplementary figures and images for: The effects of kangaroo mother care on the time to breastfeeding initiation among preterm and LBW infants: a meta-analysis of published studies
Source: Int Breastfeed J. 2019 Feb 19;14:12. doi: 10.1186/s13006-019-0206-0 (PMC6379962; doi:10.1186/s13006-019-0206-0)

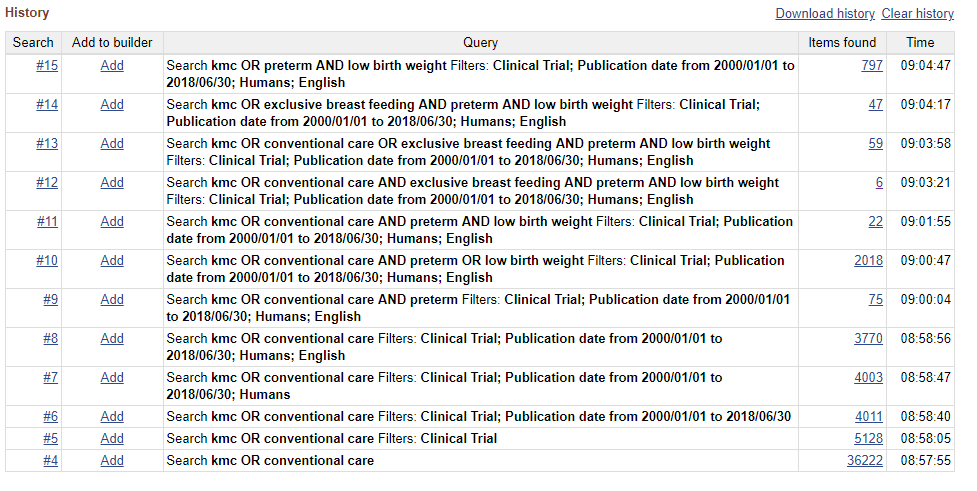


Cochrane review


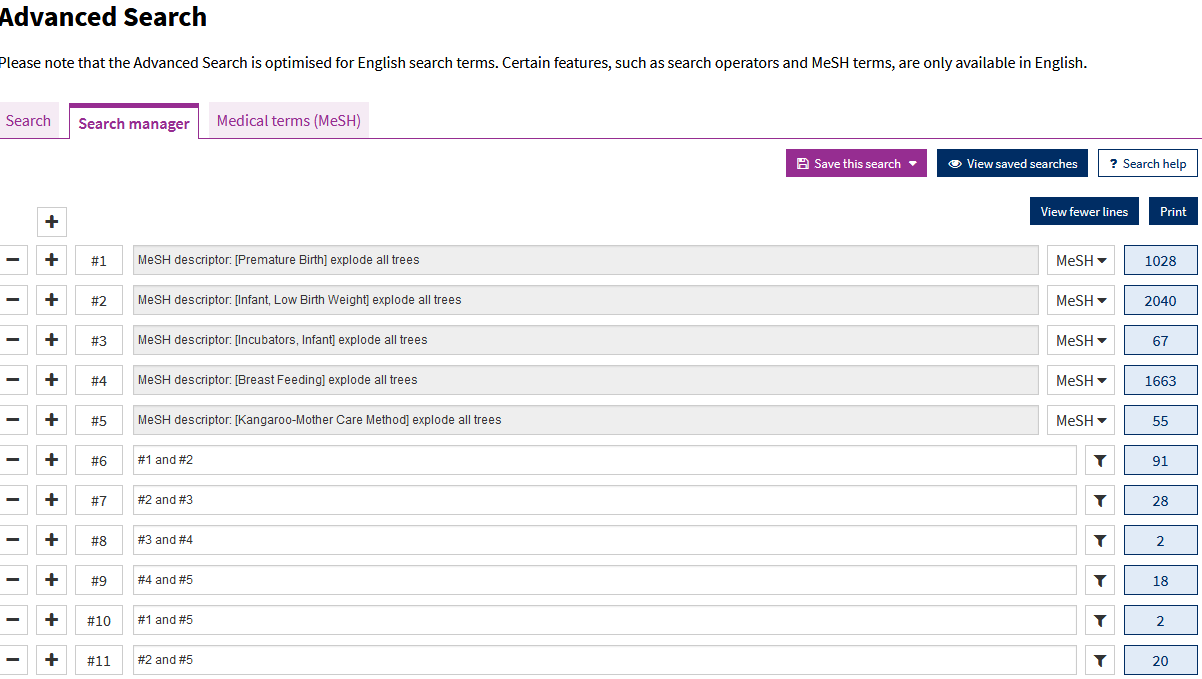

Supplement: Supplementary file 1 — MEDLINE via PubMed and Cochrane database search for randomized controlled trial studies of KMC versus conventional care method, June 30, 2018. (DOCX 150 kb) [file 13006_2019_206_MOESM1_ESM.docx]
